# Real Time Quantification of Dangerousity in Football Using Spatiotemporal Tracking Data

**DOI:** 10.1371/journal.pone.0168768

**Published:** 2016-12-30

**Authors:** Daniel Link, Steffen Lang, Philipp Seidenschwarz

**Affiliations:** Department of Exercise Science and Sport Informatics, Technical University of Munich, Munich. Germany; Northwestern University, UNITED STATES

## Abstract

This study describes an approach to quantification of attacking performance in football. Our procedure determines a quantitative representation of the probability of a goal being scored for every point in time at which a player is in possession of the ball–we refer to this as dangerousity. The calculation is based on the spatial constellation of the player and the ball, and comprises four components: (1) Zone describes the danger of a goal being scored from the position of the player on the ball, (2) Control stands for the extent to which the player can implement his tactical intention on the basis of the ball dynamics, (3) Pressure represents the possibility that the defending team prevent the player from completing an action with the ball and (4) Density is the chance of being able to defend the ball after the action. Other metrics can be derived from dangerousity by means of which questions relating to analysis of the play can be answered. Action Value represents the extent to which the player can make a situation more dangerous through his possession of the ball. Performance quantifies the number and quality of the attacks by a team over a period of time, while Dominance describes the difference in performance between teams. The evaluation uses the correlation between probability of winning the match (derived from betting odds) and performance indicators, and indicates that among Goal difference (r = .55), difference in Shots on Goal (r = .58), difference in Passing Accuracy (r = .56), Tackling Rate (r = .24) Ball Possession (r = .71) and Dominance (r = .82), the latter makes the largest contribution to explaining the skill of teams. We use these metrics to analyse individual actions in a match, to describe passages of play, and to characterise the performance and efficiency of teams over the season. For future studies, they provide a criterion that does not depend on chance or results to investigate the influence of central events in a match, various playing systems or tactical group concepts on success.

## Introduction

The availability of virtually all-encompassing positional data in professional football presents new challenges for the way in which that data is analysed and interpreted. They relate equally to analysis of games in clubs, product design for reporting in the mass media, and new analytical procedures for addressing academic questions. A significant factor in this context comprises the description of the technical-tactical aspects of the events of a match by means of performance indicators [1]. Although traditional indicators, such as shots on goal, number of passes, tackle rates, team ball possession and distances covered are widely used, their significance for performance is open to critical question [2,3]. The key task for data science and sports science is to derive intelligent indicators from raw data that describe relevant components of the game appropriately.

Recent years have seen an increasing number of publications that report successes in identifying tactical structures. Grunz, Memmert and Perl [4] use self-organising maps to classify the behaviour of small groups of players in set play situations, such as a game opening sequence. Bialkowski et al [5] present a method that can adaptively assign roles played by individual players. Similarly, playing styles can be described through the spatial distribution of plays [6] or the characterisation of ball possession phases through gains in territory, the number of passes or the speed of play [7]. From retrospective analysis of goals and shots on goal, promising spatial constellations can be classified [8] or metrics of network analysis can be used to describe the proportion of individual players involved in the team’s success [9,10].

This paper suggests a solution to a question that has largely been unresolved to date, namely: How can success in football be quantified? Until now, there has been no convincing procedure available by means of which the value of a piece of dribbling can be compared with a pass, or various passing options compared with one another. If a coach wants to know whether a change in defensive midfield has led to greater stability in defence, he has not so far had any quantitative criterion that would allow such an assessment. Conclusions about the general success of tactical measures against an opponent who is sitting deep, for example, also require a yardstick by which “more successful” can be measured.

When we use the term “success” in the sense of performance analytics, we are not referring to the outcome of the game. Goals are scored only rarely in football, and can come about through an individual moment of loss of concentration, while a very dominant team might simply be unlucky sometimes. Rather, in order to answer the question posed above, a criterion is required that allows an evaluation of the extent to which tactical objectives were achieved. We believe that creation of situations in which there is a danger of a goal being scored or the prevention of such situations for the opponent, should be the central criterion in characterising tactical success, or “performance” in football. Shots on goal may be a better criterion than goals in this context, although they may arise in situations that are not dangerous or a player may be prevented from shooting just a few metres in front of goal. In the semi-final of the 2014 World Cup, for example, Brazil had more shots on goal than Germany (18 vs. 14) [11], but hardly any observer would doubt Germany’s superiority in that match (result 1:7).

Therefore, our approach to describing success does not use events but a quantitative representation of the probability of a goal, which we describe as *Dangerousity*. We calculate this value for every moment during which a player is on the ball. Dangerousity is related to the construct “scoring opportunity”, but its quality is not evaluated by guesswork but by a defined process using an algorithm. Additionally, we derive other metrics from dangerousity by means of which questions relating to analysis of the play can be answered. *Action Value* represents the extent to which the player can make a situation more dangerous through his possession of the ball. *Performance* quantifies the number and quality of the attacks by a team over a period of time, while *Dominance* describes the difference in performance between teams.

Our modelling procedure follows the paradigms of rationalism and deduction rather than empiricism and induction [12]. In other words: our starting point is our understanding of football. We believe that dangerousity is mostly determined by four factors: (1) the position, so called *Zone* of the ball, (2) the degree of *Ball Control*, (3) the *Pressure* that is put on the player by the opponent and (4) the *Density* of opponent players in front of the goal. While there are other factors, we suggest that these four components are the key indicators. To operationalize these indicators, we use mathematical functions which take spatiotemporal data as their input. We choose their functional form in a way that fulfils our analytical understanding of the game (e.g. a defender behind an attacker creates less pressure compared to a defender in front of the attacker).

To date there have been two similar approaches in basketball [13] and in football [14]. In these, the probabilities of success are also described continuously by means of a so-called *expected position value* (EPV) or an *expected goal value* (EGV). The approach in football determines this value on the basis of position, distance from defenders and the match context (e.g. open play, counter-attack). In contrast to our procedure, which takes account of all match situations at a distance of less than 34 m from the opponent’s goal line, in EGV calculations, only the last 10 seconds before shots on goal are considered. The procedure is also based on a less explicit modelling of the individual components that make up the danger of a goal being scored. It is also worth mentioning that the company ProZone markets a construct of dangerousity [15], but details of the way it operates have not been published to academic standards.

The aim of this paper is threefold: Firstly, it shows, how dangerousity and derived metrics are quantified. Some details of the specification are left to one side; instead, the focus is on the underlying ideas and relationships. Secondly, an evaluation of the quality of the quantification is carried out, together with the quantitative evidence for the construct’s relevance to performance. In the view of the authors, these components of validation, in particular, are not sufficiently documented by many competition information providers (CIP) even though they represent a central component in the development of performance indicators in sports. To our knowledge, this paper is the first to use the correlation with betting odds as a criterion for the relevance of performance indicators. Thirdly, the paper shows examples of how the metrics developed can be used to answer questions relating to analysis of a match with different time horizons.

## Quantifying Dangerousity

### Dangerousity

*Dangerousity* (DA) is present for every moment in which a player is in possession of the ball—and can therefore complete an action with the ball. We refer to this time span as *Individual Ball Action* (IBA) and to the player concerned as the *IBA-player*. IBA exists as soon as the distance between the player and the ball falls below a threshold and the ball is then touched. IBA ends when the ball is out of the player’s range once again. The procedure is described in detail in Hoernig, Link, Herrmann, Radig, and Lames [16].

Dangerousity is based on the four components *Zone* (ZO), *Control* (CO), *Pressure* (PR) and *Density* (DE), where the first two components increase and the last two components decrease its value. Zone represents the danger of a goal being scored from the position of the IBA-player, Control stands for the extent to which the player can implement his tactical intention on the basis of the ball dynamics, Pressure represents the opportunity of the defending team to prevent the IBA-player from completing an action with the ball and Density is the chance of being able to defend the ball after the action. The value range for all of the constructs is between 0 (low) and 1 (high).

These individual components give the Dangerousity for a moment t as the product of Zone and a linear combination of Control, Pressure and Density (Eq 1). The model constant k_1_ quantifies the extent to which these three figures reduce the value for Zone. It is selected in such a way that Zone is reduced by a maximum of a factor of 0.5. As a lack of control of the ball results in a reduction in the Dangerousity, Control is included as negated.

$$DA\;(t)=ZO\;(t)*(1-\frac{1-CO\;(t)+PR\;(t)+DE\;(t)}{k_{1}})$$ (Eq. 1)

**Fig 1:**
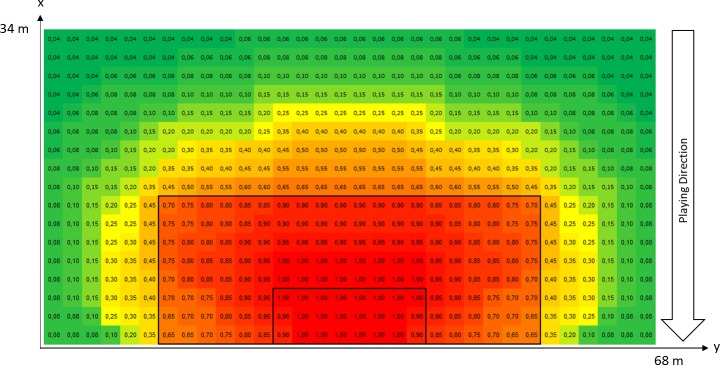
Quantification of Zone is carried out using the position of the IBA-player on a 2 x 2 m grid that begins 34 m from the goal line.

Quantification of Zone is carried out using the position of the IBA-player on a 2 x 2 m grid that begins 34 m from the goal line (Fig 1). Our evaluation of a position is based on several assumptions: First, as the distance from the goal decreases and centrality increases, the danger rises [14,17]. Second, moving into the penalty area brings about a sudden increase in the danger because of the risk of a penalty kick [18]. Third, there is a homogeneous area in front of goal in which the danger does not increase further. Fourth, an acute angle to the goal reduces the danger. Fifth, areas to the side of the penalty area are dangerous because of the possibility of a cross with little risk of offside.

Control is estimated by means of the average relative speed (v_rel_) of ball and IBA-player in the last 0.5 s. High relative speeds occur, for example, when the player shoots on goal with just brief contact with the ball after a cross. Comparatively low relative speeds are found when the player has the ball at his feet for a longer period, when dribbling, for example, or positioning the ball for a shot on goal. We believe that at relative low relative speeds, there is an almost perfect CO near a value of 1. With increasing v_rel_, it gets more and more difficult to control the ball. We model this by using a quadratic function, moderated by the model constant k_2_ (Eq 2). If v_rel_ is above 25 ms^-1^, CO is equal to 0.

$$CO\;(v_{rel})=1-k_{2}*{v_{rel}}^{2}$$ (Eq. 2)

**Fig 2:**
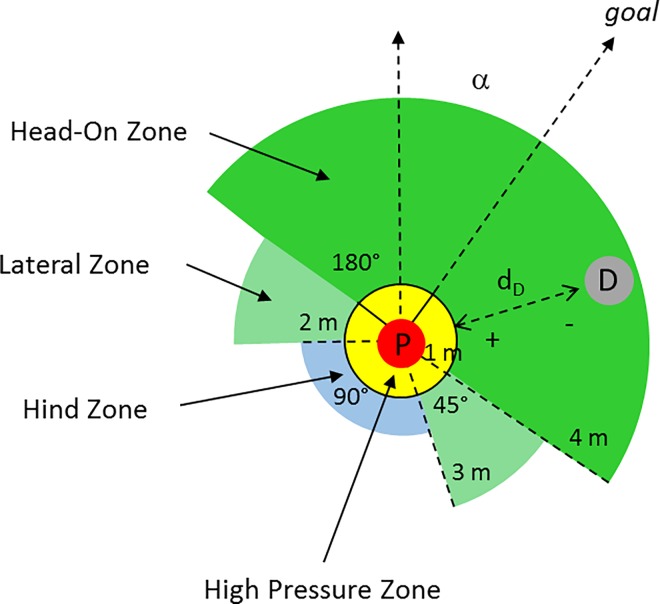
Geometry to determine Pressure. The Pressure Zone covers four sub-areas with different radii, which result from the angle (α) between IBA-player and the center of the goal. Pressure depends on the sub-area and the distance (d_D_).

In determining Pressure, we assume that a *defender* (D) exerts pressure when his distance (d_D_) from the IBA-player (P) is below a threshold value r_ZO_. The *Pressure Zone* (PZ) covers four sub-areas with different radii (r_ZO_), which result from the angle (α) between IBA-player and the centre of the goal (Fig 2). This is based on the assumption that a defender who is between the IBA-player with the ball and the goal (Head on Zone) is more likely to be able to defend a scoring opportunity than a defender who is to the side (Lateral Zone) or behind (Hind Zone). Within the zones, there is a linear increase in pressure as the distance falls. If the defender is very close to the IBA-player (High Pressure Zone), the pressure is constantly high. An individual defender (D_i_) creates Pressure (PR_Di_) in accordance with (Eq 3). Our model bases on the idea, that every additional defender increases Pressure, although the increase gets less with every additional defender. We model this using a logarithmical function, moderated by the model constant k_3_ (Eq 4).

$${PR}_{D_{i}}\;(d_{D_{i}},\;\alpha )=1-\frac{d_{D_{i}}}{r_{ZO}(\alpha )}$$ (Eq. 3)

$$PR\;\left(x\right)=1-e^{-k_{3}x},\;where\;x=\sum_{\forall \;D_{i}\;inside\;PZ}PR_{D_{i}}$$ (Eq. 4)

Density is described by means of two components: *Shot Density (SD)* represents the probability of a team blocking a shot, while *Pass Density* (PD) indicates the likelihood of intercepting an offensive pass or cross. Depending on the *Centrality* (C) of the IBA-player, the two components are weighted differently (Eq 5). At an acute angle to the goal, Pass Density is weighted higher, in a central position the Shot Density is greater.

DE(c)=C*SD+(1−C)*PD (Eq. 5)

**Fig 3:**
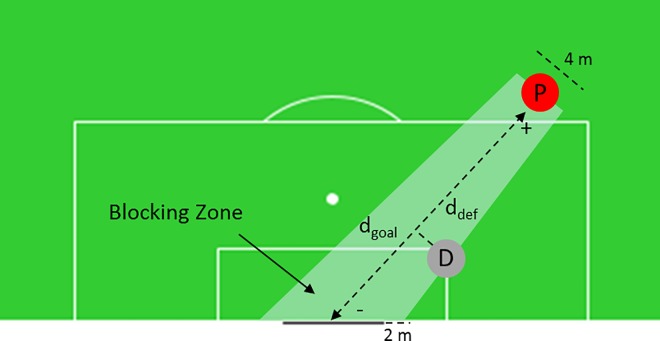
Geometry to determine Shot Density. A defender increases Shot Density if he is in the Blocking Zone formed between the position of the IBA-player and the goal. The figure is calculated from the distance between the IBA-player (P) and the goal (d_goal_) and between the IBA-player and the defender (d_D_).

A defender increases Shot Density if he is in the *Blocking Zone* (BZ) formed between the position of the IBA-player and the goal (Fig 3). The value is calculated from the distance between the IBA-player (P) and the goal (d_goal_) and between the IBA-player and the defender (d_Di_). The smaller *d*_*Di*_ is, the higher the density created by that player because a larger area of the goal is potentially covered. For a defender (D_i_), the SD_Di_ created by him is given by (Eq 6). Every additional defender within the BZ increases the density, although the increase is also attenuated logarithmically similar to (Eq 4), but with using a different model constant k_3_.

$${SD}_{D}(d_{D},\;d_{goal})=1-(d_{D})\frac{d_{D}}{d_{goal}}$$ (Eq. 6)

**Fig 4:**
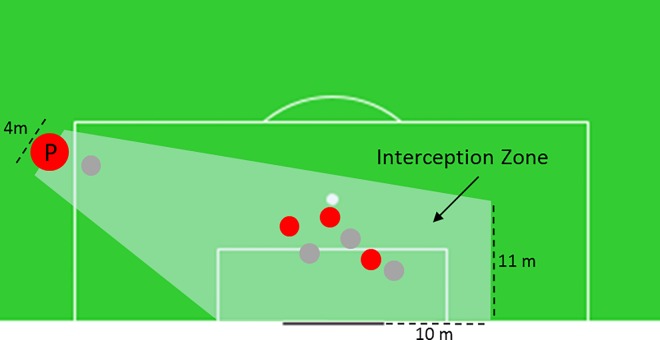
Geometry to determine Pass Density. Pass Density depends on the difference between the number of defenders and attackers (Majority) within the Interception Zone (IZ).

Pass Density depends on the difference between the number of defenders and attackers within the *Interception Zone* (IZ). We call this difference *Majority (M)*. For example, if there are 4 defenders and 3 attackers in the IZ, M is equal to 1 (Fig 4). As the Majority of defenders increases, Pass Density approaches a value of 1, with a Majority of attackers it moves towards 0. This understanding is operationalized by using an arcus tangent function (Eq 7). The model constant k_5_ describes the sensitivity of the model.

$$PD\;(M)=0.5+\frac{\tan^{-1}\;(k_{5}M)}{\pi }$$ (Eq. 7)

### Derived metrics

Dangerousity is present in the measuring frequency of the tracking system and can therefore be used to describe the value of IBAs. We call this quantity *Action Value* (AV) (Eq 8). For calculating this we use the difference between Dangerousity at the moment when a player has IBA (Start IBA) and the moment when the next player has IBA. If this is a player of the opposing team or DA decreased during the IBA, Action Value is negative.

$$AV\;({IBA}_{n})=DA\;(Start\;{IBA}_{n+1})-DA\;(Start\;{IBA}_{n})$$ (Eq. 8)

In order to assess the success of the attack, which we call *Performance* (PE), of a team over a longer period, the match is divided into intervals of 5 s in length and the maximum value for DA is determined for both teams over an interval i (DA_i_). Performance is then given by the sum of this value for all intervals over the period (*ts*), as *Match Performance* (MP), for example (Eq 9). This discretisation ensures that there are the same number of summands for both teams over a given period. Furthermore, we use *Current Performance* (CP) in order to describe the course of play over a time interval. We determine this for a moment t using past values for DA_i_ in the intervals of the last 5 minutes.

$$MP\;(ts)=\sum_{\forall i:\;{Interval}_{i}\in ts}{DA}_{i}$$ (Eq. 9)

While the previous metrics are based on an evaluation of the attacking play of a team, *Dominance* (DO) describes the difference in performance between the two teams (T1, T2). This can be calculated both over a time interval as *Match Dominance* (MD), for example, and for a moment as *Current Dominance* (CD). In both cases, this is provided by the difference in the Performance of the two teams (Eq 10).

DO(T1)=PE(T1)−PE(T2) (Eq. 10)

### Calibration

The calibration processes intended to optimizes the model constants k_1_, k_2_, k_3_, k_4_, k_5_ manually. In collaboration with football experts, a large number of different match situations were analysed in detail, such as positional attacks, counter-attacks, 1 vs. 1 situations, crosses and dribbling with the ball by individual players. With the aid of a self-developed software package, the individual match scenarios were compared with the quantification of the components in the dangerousity model. In this process it was possible to simulate the effect of changing model constants and to optimise them gradually. A total of over 100 situations were examined for apparent validity (see example in section Individual action analysis).

### Limitations

In balancing complexity, the accuracy of the positional data and the benefit for performance diagnostics, the procedure presented here does not take account of all aspects of dangerousity. These include the movement dynamics of the players and the ball, the direction in which the players are looking, their position in relation to the ball, the extent to which teammates are available [19,20] and different individual skills. Also all geometrical parameters base on our qualitative evaluation of game situations, our interpretation and—at the end—on our philosophy of the game. Further studies should empirically validate some assumptions, e.g. the model for Zone. The treatment of special cases such as standard situations, off sides and retrospective sanctions for fouls is out of the papers scope.

## Evaluation

### Reliability of measurement

This paper is based on 64 games in the German National Football League (Bundesliga) in the 2014/15 season. The positional data was collected by a CIP (TRACAB corp.) via an optical tracking system and then reviewed manually. Since each player agreed to this procedure on signing his contract of employment as a professional football player, special approval for this study from an ethics committee was not required. Nevertheless, all procedures performed in the study were in strict accordance with the Declaration of Helsinki as well as with the ethical standards of the Chair of Training Science and Sports Informatics of the Technical University of Munich.

In assessing the quality of the quantification, the automatic calculation of dangerousity was compared with the evaluation by semi-professional football coaches in 100 match scenarios. The sample was selected in such a way that the value range of DA was covered evenly. Three experts evaluated the scenarios independently of one another on the basis of video recordings using a scale of 1 (little danger) to 5 (very dangerous). They had no knowledge of the underlying model, but were asked to evaluate the scenarios qualitatively in their entirety. For the statistical analysis, we grouped the situations into *Danger Groups* (DG) following their assessments by the majority principle and checked for differences using an ANOVA.

**Fig 5:**
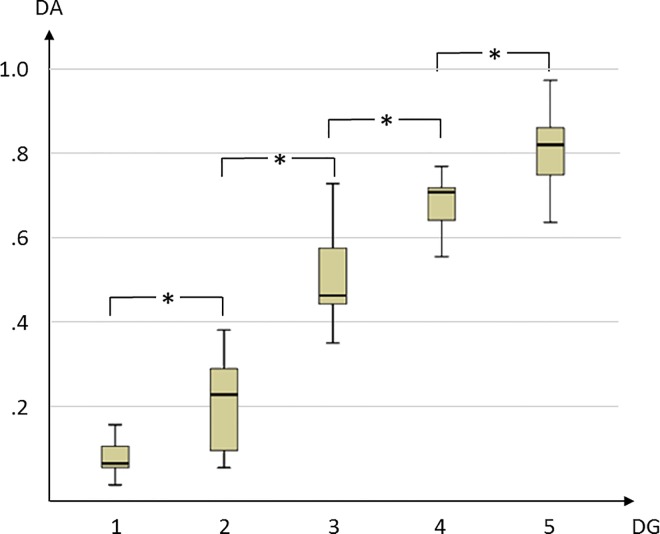
Boxplot of Danger. Match scenarios (n = 100) were grouped into Danger Groups (DG) by experts using a scale of 1 (little danger) to 5 (very dangerous). Scenarios that were classified as dangerous by the observers were also classified as dangerous on average by the algorithm. All of the post hoc tests between neighboring groups also showed significant differences (α = .01).

The results show that the mean value for DA differed significantly between the groups (F = 170.31, p < .01) (Fig 5). All of the post-hoc tests between neighbouring groups also showed significant differences (α = .01). This means that scenarios that were classified as dangerous by the observers were also classified as dangerous on average by the algorithm. In some cases, the classifications of the observers differ from one another, but also between an observer and the algorithm. There is a fair correspondence between the observers (κ = .32, [21]). This is also to be expected, as the quantification of danger also includes subjective components.

### Performance relevance

The crucial criterion for the quality of a performance indicator is that it depicts an issue that describes an important component in the performance of a sport. This is usually checked in the performance analysis in two different ways. One possibility is to determine its capacity to forecast the outcome of a match [22,23]. Since, however, relatively few goals are scored in football, as discussed in the introduction, the number of goals is usually only moderately related to performance indicators (Table 1). A more promising approach is therefore to assess the contribution of an indicator to clarification of performance–i.e. the ongoing playing strength of a team–and to compare this with other indicators [24,25].

**Table 1:** Correlations between performance indicators and between performance indicators and skill indicator (win probability (WP) based on betting odds). The greatest correlation between WP and the performance indicators exists with Match Dominance (MD).

	skill indicator	performance indicator					
	WP	G	SG	PA	TR	BP	DOM
G	.55	x	.44	.33	.35	.34	.41
SG	.58	.44	x	.64	.20	.61	.83
PA	.56	.33	.64	X	.26	.93	.78
TR	.24	.35	.20	.26	x	.19	.14
BP	.71	.34	.61	.93	.19	x	.76
MD	.82	.41	.83	.78	.14	.76	x

For the matches in the sample, the variables *Goal* (G), *Shot at Goal* (SG), *Passing Accuracy* (PA), *Tackling Rate* (TR), *Ball Possession* (BP) and *Match Dominance* (MD) were collected. G, SG and PA represent the difference in the variable value from that of the opponent; the remaining variables already represent relative values between the teams. As an external criterion for the difference in performance between the teams, the *Win Probability* (WP) of a team was used, based on the odds from 13 bookmakers (www.Football-Data.co.uk). In the statistical analysis, the correlation coefficients for all the pairs of variables were calculated.

The highest correlation between WP and the performance indicators exists with MD (r = .82), followed by BP, SG, PA, G and TR (Table 1). Of the indicators studied, dominance is therefore the one with the highest correlation with the performance of a team. It follows from this that the probability of a stronger team in a match achieving a higher dominance is greater than the probability of it scoring more goals, for example. This is easy to explain in terms of content, as a team that is weaker in a match is more likely to score a goal from an individual situation than it is to generate more dangerous situations over the entire course of a game. In this context, the sequence given above can be taken as a way of sorting the indicators according to their relevance for match performance. The validity of dominance and therefore, in turn, of performance can thus be demonstrated both rationally and empirically-quantitatively.

Other evidence of validity emerges from the correlation of performance indicators with one another. Here the results seem entirely plausible: Shots on goal are completed mainly in situations with a high dangerousity, so there is a strong correlation between MD and SG (r = .83). As Dangerousity presupposes possession of the ball, MD and BP also show a strong correlation (r = .76). Possession of the ball and passing accuracy are almost entirely identical (r = .92). This is easy to understand because a poor pass leads directly to the loss of the ball.

## Game Analysis

### Individual action analysis

**Fig 6:**
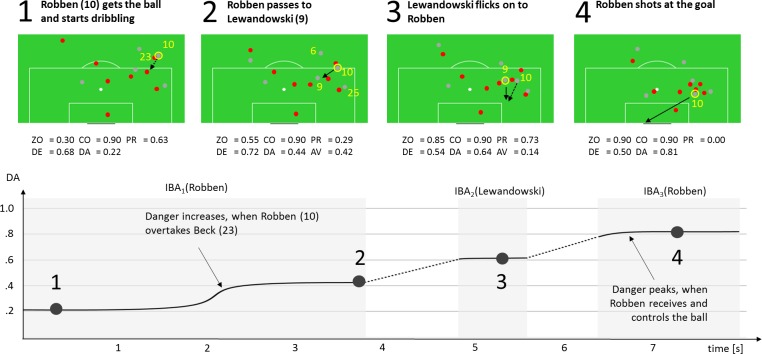
Course of Danger in a match scenario. Spatial configuration and value of model components are shown in four key moments.

A possible application at the micro level is the analysis of small sections of game situations. As dangerousity is calculated for every frame, the value changes continuously during an IBA interval. Key situations such as outplaying an opponent in an important duel or a successful pass through the defensive line cause a big jump in DA, while periods without gaining ground lead to an even signal sequence. This can be illustrated by an attack by Bayern Munich (FCB) against TSG 1899 Hoffenheim from the Saison 2014/2015 (Fig 6).

The attack comprises three IBA intervals by the players Robben (no. 10), Lewandowski (no. 9) and Robben again. At the moment when Robben takes possession of the ball, there is moderate dangerousity (DA = .22). The player begins to dribble against defender Beck (no. 23) and he succeeds in beating his opponent. This results in a reduction in PR, as Beck falls back from the High Pressure Zone into the Hind Zone. At the same time, ZO increases because of the shorter distance to the goal. At the end of IBA_1_, there are three realistic play options for Robben, apart from continuing to dribble. The pass options to Müller (no. 25) and Lewandowski would be evaluated almost equally by the Action Value (AV_IBA_1_ = .44 and .42), assuming that the spatial configuration does not change significantly, while the back pass to Thiago (no. 6) would result in a negative evaluation (AV_IBA_1_ = .19). Robben decides to pass to Lewandowski and gets the ball straight back from the player (AV_IBA_2_ = .14). After contact with the ball to take possession of it, DA increase to its maximum value in this scenario (DA = .81). There is then a stand-alone shot on goal by Robben, but from a relatively acute angle in front of the goalkeeper.

The diagnostic benefit in terms of performance of this analysis lies less in the evaluation of the playing behaviour in individual scenarios. This would require a multitude of other factors to be taken into consideration, such as movement dynamics, passing risk and individual skills, which could only be derived from a qualitative analysis of the film material. By contrast, it would be possible to evaluate the contribution of a player to the attacking play of his team over a longer period. It is possible that players will thus be identified who, although they have a lot of contact with the ball, contribute only little to increasing dangerousity in attacking phases.

The key application for the game analysis lies in filtering video material on the basis of dangerousity. Sudden increases can be understood as disruptions or perturbations in the balance between defence and attack in line with the theory of dynamic systems [26,27]. The specific selection of these scenarios, possibly in combination with other attributes such as the side of the pitch or the involvement of certain players, can simplify game analyses significantly.

### Single match analysis

Traditional match statistics provide inadequate information to assess the course of a match correctly [2,14]. As already shown in the introduction using the example of BRA—GER, shots on goal are not very suitable as a criterion for assessing performance or success in specific cases. The same applies to possession of the ball: teams that are in the lead often change their tactics and then have less possession of the ball than if they are behind [28,29]. Tackle and pass rates show only small links with the performance of teams ([22,23]; see Table 1). We therefore believe that performance or dominance allow a significantly better assessment of whether a team has been “lucky” and won through an individual action or has been able to set up many dangerous situations and has “earned” the win.

**Fig 7:**
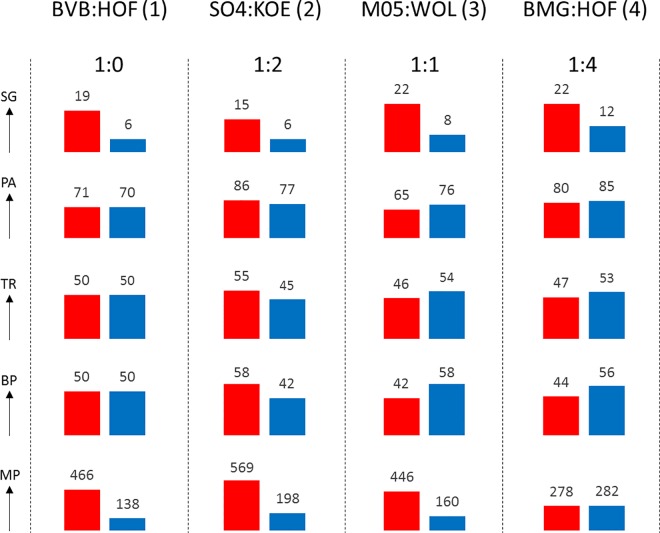
Performance indicators of 4 matches in 2014/15 Bundesliga season. Goals (G), Shots at Goal (SG), Passing Accuracy (PA), Tackling Rate (TR) and Ball Possession (BP) provide inadequate information to assess the course of a match correctly. Match Performance (MP) allow a significantly better assessment of whether a team has been “lucky” and won through an individual action or has been able to set up many dangerous situations and has “earned” the win.

Match 1 (Fig 7) provides an example of a merited victory by the home team. Here Dortmund (BVB) creates significantly more dangerous situations (PE 466:138), despite even possession of the ball. The opposite course of events can be assumed in match 2: Schalke (S04) dominates the match with a PE of 469:198, but suffers a defeat by 2:1. In match 3, although Wolfsburg (WOL) has more possession, it creates significantly lower PE from it than Mainz (M05). This constellation suggests a large number of unsuccessful positional attacks by a team that also has problems preventing their opponents from counter-attacking. In match 4, Gladbach (BMG) has more shots on goal than its opponent but without achieving an advantage in PE to the same degree. The shots on goal may have come from situations involving comparatively little danger. As far as pass and tackle rates are concerned, we do not believe that any plausible relationships are evident.

**Fig 8:**
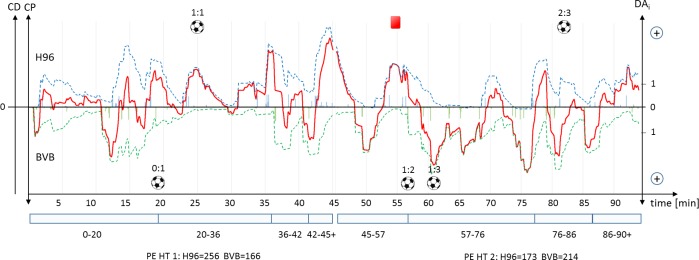
Performance variables in the course of the match Hannover (H96) vs. Dortmund (BVB). Danger for an interval (DA_i_) is visualized by bars, Current Performance (CP) by dashed lines and Current Dominance (CD) by a solid line. DA_i_ and CP were inverted for the away team. CD is shown from the perspective of the home team.

Using the metrics Current Performance and Current Dominance, success can be assessed not only for a complete match, but also for periods of time. In this way, the effects of tactical interventions (substitutions, system changes) or central events in a match can be investigated, for example. Fig 8 shows the course the match between Hannover 96 (H96) and Borussia Dortmund (BVB) on the 26^th^ match day. With the metrics, we manually identified eight key phases (P). Until the first goal is scored (0:1, 19^th^ min), the play can be described as very even (0 – 20^th^ min). Then a phase of dominance (20^th^ - 36^th^ min) began for H96, during which they took the score to 1:1 (25^th^ min). Between the 36th and 42nd minutes, BVB managed only the occasional attack. In the last 4 min of the first half, H96 clearly dominates play but without scoring another goal.

At the start of the second half (45^th^- 57^th^ min), play was very even up to the sending-off for H96 (55^th^ min), with a slight advantage to H96. The dominant phase for BVB (57^th^-76^th^ min) began between their second goal (1:2, 57^th^ min) and their third (1:3, 61^st^ min). This may have been the result of their superiority in numbers and/or psychological elements. This phase ended around 15 minutes before the end of the match. After conceding their second goal (2:3, 82^nd^ min), BVB recorded no further successful attacks and obviously attempted to play out time. H96 was able to create a further series of dangerous plays, but without levelling the score. If dominance is considered over the two halves, it is clear that the home team was dominant after the first half (DO = 90), while the away team had the advantage in the second half (DO = -41).

### Team efficiency ratings

At the macro level, performance and dominance are appropriate for characterising the performance of the team as a whole. It is important firstly to consider the attacking performance and the defensive performance (as the inverse of the opponent’s attacking performance) over a large number of matches and to put these in the context of the other teams. This is a good starting point for describing team efficiency, which can be defined as the relation between points achieved and the dominance. This allows to answer the question of the extent to which the examples of lucky victories shown in Fig 8 are balanced out by unlucky defeats over the course of a season, for example.

**Fig 9:**
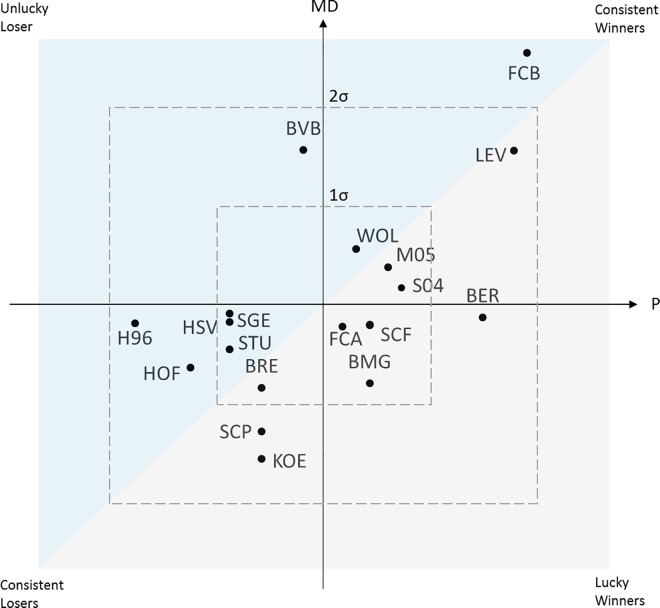
Ranking of teams based on 64 matches. The vertical position of a team indicates its average Match Dominance (MD), the horizontal position describes the number of points (P) it has gained. The quadrants classify team according to the factors successful vs. less successful and consistent vs. lucky. A position above the main diagonal indicates an unfavorable ratio of Dominance to success (Points), a position below it a positive ratio.

Even though we have only 64 matches at our disposal for our study, the principle of the procedure can be illustrated in Fig 9. Here the vertical position of a team indicates its average dominance, the horizontal position describes the number of points it has gained. In the 1^st^ quadrant there are teams that both dominated in matches and have had above-average success. Teams in the 2^nd^ quadrant dominated but were less successful. Similarly, the subordinate teams in terms of play can be divided into successful (3^rd^ quadrant) and unsuccessful (4^th^ quadrant).

If one interprets the main diagonal as the number of points expected for the level of dominance, the horizontal distance of a team from this diagonal can be understood as the discrepancy between effort and success. A position above the main diagonal indicates an unfavourable ratio of dominance to success, a position below it a positive ratio. In other words, the teams below the diagonal were quite efficient, the teams above were not. In the matches examined, for example, the teams from Dortmund (BVB) and Hanover (H96) were significantly less successful than they “deserved” to be based on their dominance. Berlin (BER), Gladbach (BMG) and Cologne (KOE), on the other hand, can be happy with their points return in view of their match performance. The success of Leverkusen (LEV) and Mainz (M05) roughly corresponds to their match performance.

## Conclusion

The aim of the study was to develop, evaluate, and apply a procedure for determining dangerousity in football with real-time capability. The evaluation showed that the quantification of this construct using the spatial constellation of players and ball lies in the same range as human observers. Individual misinterpretations play only a subordinate role in the validity of diagnostic findings concerning performance, particularly with large data volumes. Like many other tactical elements in football, however, the construct does contain a certain lack of precision, with the result that a clear reference for the accuracy of the measurement in the sense of a ground truth cannot exist.

The performance and dominance metrics derived are more robust in the context of the effects of chance, and map the match performance of a team more reliably than the traditional performance indicators of possession of the ball, shots on goal, tackle, and pass rates. They can be used to evaluate individual plays, to describe efficiency, represent passages of play, or compare players and teams with one another. In particular, they can help to investigate questions relating to the influence of various playing systems or tactical group concepts on success. In addition, the metrics can be used as the basis for the development of media products, e.g. the fever curve in Fig 8 could be shown during television broadcastings, live event tickers, or second screen applications.
